# Removal of albumin and immunoglobulins from canine cerebrospinal fluid using depletion kits: a feasibility study

**DOI:** 10.1186/2045-8118-11-14

**Published:** 2014-06-23

**Authors:** Ramona Günther, Eberhard Krause, Michael Schümann, Jérome Ausseil, Jean-Michel Heard, Ingolf E Blasig, Reiner F Haseloff

**Affiliations:** 1 Leibniz-Institut für Molekulare Pharmakologie, Robert-Rössle-Str. 10, 13125 Berlin, Germany; 2 Laboratoire de Biochimie, Centre de Biologie Humaine, Hôpital Sud, CHU Amiens, Avenue René Laënnec, 80054 Amiens and Unité INSERM U1088, Pôle Santé, Université de Picardie Jules Verne, Rue des Louvels, 80000 Amiens, France; 3 Institut Pasteur, 25-28 rue du Dr Roux, 75724, Paris, Cedex 15, France

**Keywords:** Cerebrospinal fluid, Dog, Depletion, Albumin, Mass spectrometry

## Abstract

**Background:**

Highly abundant proteins in biological fluids such as serum or cerebrospinal fluid (CSF) can hinder the detection of proteins in lower abundance, *e.g.*, potential biomarkers. Commercial products are available for the depletion of albumin and immunoglobulins (Igs), although most of these kits have not been validated for dog samples. The present study therefore examines the use of different types of depletion kits for dog CSF.

**Findings:**

Three kits, with different mechanisms for the depletion of albumin and Igs, were tested with dog CSF specimens. One product significantly decreased the amount of albumin; with all kits, IgG was less efficiently removed than albumin. Mass spectrometry of the fractions eluted from the depletion columns revealed considerable co-depletion of other CSF proteins.

**Conclusions:**

A commercially available depletion kit was identified which depletes albumin and (to a lower extent) immunoglobulins from dog CSF. However, the limited efficacy and the concomitant loss of other proteins from the sample should be taken into account when using this product.

## Findings

### Introduction

Biomarkers for an individual biological condition can be of outstanding value for mapping a normal or pathophysiological process or for studying the response to a therapeutic intervention. Specific proteins are of particular interest as biomarkers, as their concentrations can usually be measured cheaply and reliably. Moreover, the enormous progress in the development of proteomic techniques provides highly-specific and sensitive technologies for the identification of individual proteins, including potential biomarkers [1].

Consequently, the protein profiling of body fluids is of great interest for the identification of biomarker candidates. Albumin and immunoglobulins (Igs) are the main protein constituents of plasma or serum and other body fluids. In particular, it has been shown that albumin contributes up to 60% of the protein content of cerebrospinal fluid (CSF), which is considerably more than in plasma [2]. However, potential biomarkers are usually proteins of lower abundance and these are often easier to detect after depletion of highly-abundant species. Various procedures for depleting albumin and/or Igs have been described in the literature (*e.g.*, [3-9]), and diverse “depletion kits” can be obtained from different commercial sources which, in turn, have been tested for several parameters (*e.g.*, [4,10,11]).

Unfortunately, these depletion kits have been tested for only a limited number of donor species (usually human beings and rodents). On the other hand, there is increasing interest in the development of dog models for diseases, such as the Hurler syndrome [12], the Sanfilippo syndrome [13] or Duchenne muscular dystrophy [14]. This interest, together with the first proteomic studies directed at the identification of CSF biomarkers for canine diseases [15,16], prompted us to investigate the applicability of commercial depletion kits for dog CSF.

### Materials and methods

CSF samples from eight healthy adult Beagle dogs, four male and four females, obtained by lumbar puncture, were purchased from Seralab (Haywards Heath, UK). The provider guarantees that the specimens were collected at USDA inspected and approved facilities. These samples (pooled pairwise - one male, one female) were centrifuged (2,000 × g for 10 min) to remove cells or other insoluble substances. Proteins were precipitated overnight at 20°C by adding four sample volumes of ice-cold acetone. The proteins were pelleted by centrifugation (10,000 × g, 30 min, 4°C). The supernatant was removed and the pellet was washed with ice-cold acetone (90%). The samples were vortexed and centrifuged again (10,000 × g, 30 min, 4°C). The supernatant was discarded and the pellets were dried at room temperature (30 min). Control experiments using dog serum were performed (see Additional file 1) to confirm the compatibility of this procedure, which has been recommended for proteomic investigations using CSF [17]. The ProteoSeek™ Albumin/Ig Removal Kit (Thermo-Fisher, Bonn, Germany) was applied for albumin and Ig depletion, as based on an affinity resin column. According to the manufacturer, this kit has been positively tested with human, monkey, swine and rabbit samples. Depletion experiments directed at albumin and Ig were also carried out using the ProteoExtract Albumin Removal Kit (Merck Millipore, Darmstadt, Germany, tested by the producer for human, rabbit, rat, mouse, pig and bovine samples, with decreasing activity) and by the antibody-based Vivapure Anti-HSA/Ig Kit (Sartorius, Göttingen, Germany), designed for humans, but also reactive in rat and mouse samples (according to the product information). Proteins obtained from at least three different pairwise pooled CSF samples were applied to each kit according to the manufacturer’s instructions after acetone precipitation. Optimisation procedures suggested by the manufacturers (such as prolonged and/or repeated sample application, extended mixing) did not clearly improve the depletion results for the kits when tested in dog serum. The protein quantities subjected to depletion (100 μg each) were always lower than the upper limits specified by the respective instructions, by a factor of at least 5. Elution from the depletion columns was performed as suggested by manufacturer’s protocols (if available) or by using Laemmli buffer. Flow-through (FT) and elution fractions (E) of CSF were reconstituted in Laemmli buffer. Cysteine residues were reduced using dithiothreitol (10 mM, 15 min, 60°C) and alkylated (55 mM iodoacetamide, 15 min, 20°C) in the dark. All fractions (FT and E of each kit) were separated electrophoretically on precast Novex Tris/Glycine 4%-20% gels (Life Technologies, Darmstadt, Germany). Proteins were visualised using Coomassie Brilliant Blue G-250, and the resulting band patterns obtained from at least three different samples were qualitatively equivalent for the respective kit. All clearly stained bands of eluate fractions (originating from not less than two independent samples for each kit) were excised for subsequent mass spectrometry (MS). After in-gel digestion of proteins and peptide extraction [18], MS was conducted on a LTQ-Orbitrap XL mass spectrometer (Thermo Scientific) equipped with a reverse-phase capillary liquid chromatography system (Eksigent 2D nanoflow LC, Axel Semrau GmbH, Sprockhövel, Germany). The generated peak lists and the MASCOT server (version 2.2, Matrix Science Ltd., London/UK) were used to search against the non-redundant NCBI database (version 131022) restricted to mammals (1,974,848 sequences). A maximum of two missed cleavages was allowed, and the mass tolerance of precursor and sequence ions was set to 10 ppm and 0.35 Da, respectively. The program Scaffold (version 4.2.1, Proteome Software Inc., Portland OR, USA) was used to validate MS/MS-based peptide and protein identifications, which were accepted if established at greater than 99.0% probability with at least two identified peptides.

### Results and discussion

Different depletion kits for albumin and Igs were tested using dog CSF samples. Numerous other products for depletion are commercially available [19]. However, data providing an assessment of depletion kits is sparse even for human CSF, and several authors have found that these depletion kits give relatively low reproducibility. Previous work has shown that more than 95% albumin was depleted from human CSF with the ProteoSeek kit, resulting in a 20% increase in the number of detected proteins (from 135 to 163) [20]. The Vivapure anti-HSA/IgG kit was applied to human CSF and depleted albumin less effectively than the Sigma anti-HSA/IgG column or the ProteaPrep HSA/IgG column [21]. The Vivapure kit was found to be superior to the ProteoExtract kit for the analysis of the recovery of the prostate-specific antigen in human serum samples [22]. On the other hand, in studies on human serum, ProteoExtract has been found to give satisfactory reproducibility and specificity [23]. Other authors (*e.g.*, [24]) used the ProteoExtract kit for depleting human CSF, without reporting problems.

**Figure 1:**
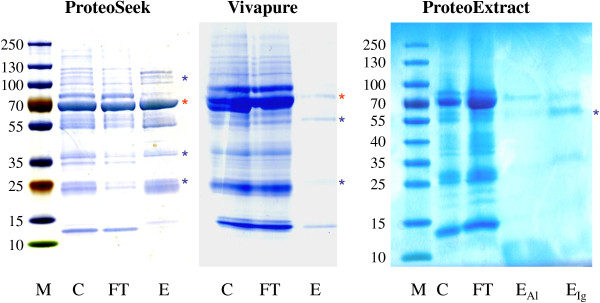
**Polyacrylamide gel electrophoresis of Coomassie blue-stained protein fractions obtained by applying different depletion kits for removal of albumin and immunoglobulins from dog cerebrospinal fluid.** Protein loads: ProteoSeek, 3 μg each; Vivapure, 3 μg (C, FT), complete eluate fraction (E); ProteoExtract, 3 μg (C) 5 μg (FT), complete eluate fractions (E_A_/E_Ig_); red/blue asterisks, mass spectrometric identification of albumin/immunoglobulin fragments; M: marker; C: dog cerebrospinal fluid (undepleted fraction); FT: flow-through fraction after depletion procedure; E: eluate fraction(s) obtained from depletion columns.

The Coomassie-stained gels shown in Figure 1 demonstrate that none of the kits gave satisfactory depletion of albumin and Igs from dog CSF samples. The ProteoSeek kit clearly exhibited the highest efficacy with respect to albumin; the amounts depleted from CSF by Vivapure and ProteoExtract kits were lower or negligible, respectively. Proteins identified in bands of the eluate gel lanes are given in Table 1 which lists proteins with substantial abundance, *i.e.*, exponentially modified protein abundance index (emPAI) > 1.0. For approximately 56% of the canine proteins found, at least one isoform coincides with the human CSF proteome previously reported [25]. Albumin was enriched in the eluate fractions obtained from the ProteoSeek and Vivapure kits, but only one low abundant fragment was found in the eluate from the ProteoExtract kit (see Additional file 2 showing all eluted proteins identified by mass spectrometry). In agreement with the protein staining, immunoglobulin fragments were detected with lower emPAI values in the eluate fractions obtained by application of the depletion kits. However, additional proteins were identified by MS in all these fractions. Significant quantities of haemoglobin (obviously a contamination of the CSF samples) were found in the eluate fractions of all kits tested. As expected from the gel staining, the greatest co-depletion was observed for the ProteoSeek kit (115 proteins detected in total), but numerous non-targeted proteins have been also identified in samples obtained with the Vivapure kit (total proteins, 64), whereas only 3 detectable proteins were eluted from the columns of the ProteoExtract kit (see Additional file 2. Table showing all eluted proteins identified by mass spectrometry).

**Table 1:** **Proteins identified by mass spectrometry with exponentially modified protein abundance index (emPAI) > 1.0**^**1**^

**Protein**	**Accession No.**^**2**^	**M.W.**^**3**^	**emPAI**_**max**_^**4**^
*ProteoSeek Depletion Kit*			
Serum albumin precursor [Canis lupus familiaris]	gi|55742764	69	11.85
Hemoglobin subunit beta-like [Canis lupus familiaris]	gi|399567834	16	6.30
PREDICTED: Serum albumin isoform X1 [Canis lupus familiaris]	gi|545520919	69	4,41
PREDICTED: Tetranectin [Canis lupus familiaris]	gi|545535577	18	4.28
PREDICTED: Apolipoprotein A-I [Canis lupus familiaris]	gi|73955106	30	3.34
PREDICTED: apolipoprotein E isoform X5 [Canis lupus familiaris]	gi|545488191	47	3.20
Immunoglobulin heavy chain variable region [Mus musculus]	gi|81237613	13	2.90
PREDICTED: Complement component C7 isoform 1 [Canis lupus familiaris]	gi|73953824	95	2.21
PREDICTED: Immunoglobulin lambda-like polypeptide 5-like isoform X1 [Canis lupus familiaris]	gi|545544681	25	2.12
Prostaglandin-H2 D-isomerase precursor [Canis lupus familiaris]	gi|50978842	21	2.08
Hemoglobin subunit alpha [Canis lupus familiaris]	gi|44888810	15	1.96
PREDICTED: Immunoglobulin lambda-like polypeptide 5-like isoform X2 [Canis lupus familiaris]	gi|545544683	25	1.77
PREDICTED: Gelsolin [Canis lupus familiaris]	gi|545518174	94	1.70
PREDICTED: Fibulin-1 isoform X1 [Canis lupus familiaris]	gi|545514459	78	1.63
Immunoglobulin gamma heavy chain A [Canis lupus familiaris]	gi|17066524	52	1.63
Immunoglobulin gamma heavy chain B [Canis lupus familiaris]	gi|17066526	52	1.61
PREDICTED: EGF containing fibulin-like extracellular matrix protein 1 isoform X3 [Canis lupus familiaris]	gi|57092953	55	1.60
PREDICTED: Angiotensinogen [Canis lupus familiaris]	gi|545494757	52	1.53
Immunoglobulin heavy chain constant region CH2 [Canis lupus familiaris]	gi|124390009	12	1.45
Ig heavy chain V region GOM [Canis lupus familiaris]	gi|123768	12	1.37
Immunoglobulin heavy chain constant region CH4 [Canis lupus familiaris]	gi|124390013	14	1.21
PREDICTED: LOW QUALITY PROTEIN: Serotransferrin isoform 1 [Canis lupus familiaris]	gi|545539001	85	1.16
Protease serine 4 isoform B [Homo sapiens]	gi|33126535	28	1.13
Clusterin precursor [Canis lupus familiaris]	gi|50979240	52	1.08
PREDICTED: Plasminogen isoform X1 [Canis lupus familiaris]	gi|545485785	91	1.06
*Vivapure Depletion Kit*			
Serum albumin precursor [Canis lupus familiaris]	gi|55742764	69	11.30
Hemoglobin subunit beta-like [Canis lupus familiaris]	gi|399567834	16	5.69
PREDICTED: Apolipoprotein A-I [Canis lupus familiaris]	gi|73955106	30	4.80
PREDICTED: Angiotensinogen [Canis lupus familiaris]	gi|545494757	52	3.35
PREDICTED: Serum albumin isoform X1 [Canis lupus familiaris]	gi|545520919	69	2.98
Hemoglobin subunit alpha [Canis lupus familiaris]	gi|44888810	15	2.91
Immunoglobulin gamma heavy chain B [Canis lupus familiaris]	gi|17066526	52	2.58
PREDICTED: Immunoglobulin lambda-like polypeptide 5-like isoform X1 [Canis lupus familiaris]	gi|545544681	25	1.62
PREDICTED: Immunoglobulin lambda-like polypeptide 5-like isoform X2 [Canis lupus familiaris]	gi|545544683	25	1.62
Prostaglandin-H2 D-isomerase precursor [Canis lupus familiaris]	gi|50978842	21	1.33
Albumin, isoform CRA_h [Homo sapiens]	gi|119626071	69	1.21
Immunoglobulin heavy chain constant region CH2 [Canis lupus familiaris]	gi|124390009	12	1.18
Immunoglobulin gamma heavy chain A [Canis lupus familiaris]	gi|17066524	52	1.16
beta-2-Glycoprotein 1 precursor [Canis lupus familiaris]	gi|54792721	38	1.00
*ProteoExtract Depletion Kit*			
Immunoglobulin heavy chain variable region [Mus musculus]	gi|81237613	13	2.39
Hemoglobin subunit alpha [Canis lupus familiaris]	gi|122508	15	1.64

^1^The complete list of identified proteins is available at Additional file 2.

^2^Accession number in NCBI database.

^3^M.W., Protein molecular weight in kDa.

^4^The emPAI value expresses mass spectrometry-based quantitation of protein abundance and is calculated according to $\mathrm{emPAI}=10^{\frac{N_{\mathrm{observed}}}{N_{\mathrm{observable}}}}-1$, (*N*_*observed*_*, N*_*observable*_, number of observed/observable peptides); cf. http://www.matrixscience.com/help/quant_empai_help.html for more details.

Unspecific binding, either to the matrix or to the bait material, is a well-known phenomenon occurring in affinity purification approaches. In addition, one has to expect that proteins bound to albumin will recover in the eluate fractions. All of the proteins identified in the eluate were also present in the recently characterised CSF “albuminome” [21] for ProteoExtract, compared to 44% for Vivapure and 30% for ProteoSeek, although this comparison does not consider different experimental conditions and species. Since albuminome data were obtained in human CSF samples, it is likely that the co-depletion observed in our experiments results from both albumin binding and unspecific association.

In conclusion, our results do not support the use of these kits for depletion of albumin and Igs from dog CSF samples. Even with the most active kit tested, albumin and Igs were only incompletely removed. This was accompanied by co-depletion of numerous other proteins, a particular drawback for the identification of potential biomarkers.

## Abbreviations

CSF: Cerebrospinal fluid; E: Elution; emPAI: Exponentially modified protein abundance index; F: Flow-through; Ig: Immunoglobulin; MS: Mass spectrometry.

## Competing interests

The authors declare that they have no competing interests.

## Authors’ contributions

RG carried out the biochemical experiments and participated in drafting the manuscript. EK and MS planned and performed mass spectrometric experiments. JA, JH and IB participated in the design of the study, the evaluation of the data and in drafting the manuscript. RH conceived the study and participated in its design and coordination and in drafting the manuscript. All authors read and approved the final manuscript.

## Supplementary Material

Additional file 1 **Gel electrophoresis of dog serum before and after albumin depletion using the ProteoSeek kit.** The flow-through fraction and the column eluate were compared with and without prior acetone precipitation. Click here for file

Additional file 2 Table showing all eluted proteins identified by mass spectrometry. Click here for file
