# Supplementary material for: Removal of albumin and immunoglobulins from canine cerebrospinal fluid using depletion kits: a feasibility study
Source: Fluids Barriers CNS. 2014 Jun 23;11:14. doi: 10.1186/2045-8118-11-14 (PMC4079625; doi:10.1186/2045-8118-11-14)
Supplement: Additional file 1 — Gel electrophoresis of dog serum before and after albumin depletion using the ProteoSeek kit. The flow-through fraction and the column eluate were compared with and without prior acetone precipitation. [file 2045-8118-11-14-S1.ppt]

## Slide 1
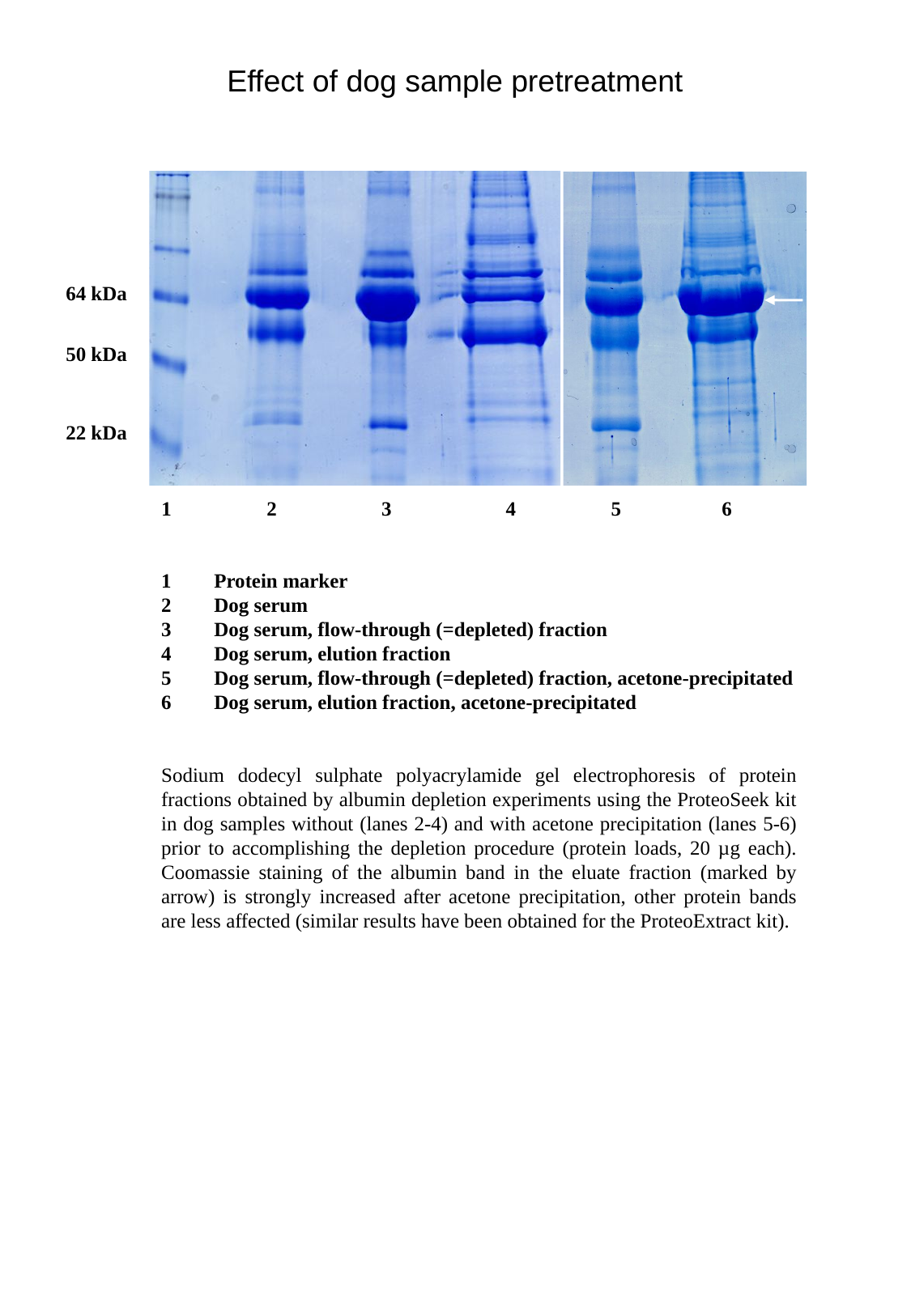

Effect of dog sample pretreatment
64 kDa
50 kDa
22 kDa
1
2
3
4
5
6
	Protein marker
	Dog serum
	Dog serum, flow-through (=depleted) fraction
	Dog serum, elution fraction
	Dog serum, flow-through (=depleted) fraction, acetone-precipitated
	Dog serum, elution fraction, acetone-precipitated
Sodium dodecyl sulphate polyacrylamide gel electrophoresis of protein fractions obtained by albumin depletion experiments using the ProteoSeek kit in dog samples without (lanes 2-4) and with acetone precipitation (lanes 5-6) prior to accomplishing the depletion procedure (protein loads, 20 µg each). Coomassie staining of the albumin band in the eluate fraction (marked by arrow) is strongly increased after acetone precipitation, other protein bands are less affected (similar results have been obtained for the ProteoExtract kit).
