# Supplementary material for: Removal of albumin and immunoglobulins from canine cerebrospinal fluid using depletion kits: a feasibility study
Source: Fluids Barriers CNS. 2014 Jun 23;11:14. doi: 10.1186/2045-8118-11-14 (PMC4079625; doi:10.1186/2045-8118-11-14)
Supplement: Additional file 2 — Table showing all eluted proteins identified by mass spectrometry. [file 2045-8118-11-14-S2.pdf]

Additional Table 1: Proteins identified in eluate fractions of different depletion kits

Protein	Accession[1]	M.W.[2]	$emPAI_{max}$[3]
ProteoSeek			
Serum albumin precursor [Canis lupus familiaris]	gi|55742764	69	11.85
Hemoglobin subunit beta-like [Canis lupus familiaris]	gi|399567834	16	6.30
PREDICTED: Tetranectin [Canis lupus familiaris]	gi|545535577	18	4.28
PREDICTED: Serum albumin isoform X1 [Canis lupus familiaris]	gi|545520919	69	4.41
PREDICTED: Apolipoprotein A-I [Canis lupus familiaris]	gi|73955106	30	3.34
PREDICTED: Apolipoprotein E isoform X5 [Canis lupus familiaris]	gi|545488191	47	3.20
Immunoglobulin heavy chain variable region [Mus musculus]	gi|81237613	13	2.90
PREDICTED: Complement component C7 isoform 1 [Canis lupus familiaris]	gi|73953824	95	2.21
PREDICTED: Immunoglobulin lambda-like polypeptide 5-like isoform X1 [Canis lupus familiaris]	gi|545544681	25	2.12
Prostaglandin-H2 D-isomerase precursor [Canis lupus familiaris]	gi|50978842	21	2.08
Hemoglobin subunit alpha [Canis lupus familiaris]	gi|44888810	15	1.96
PREDICTED: Immunoglobulin lambda-like polypeptide 5-like isoform X2 [Canis lupus familiaris]	gi|545544683	25	1.77
PREDICTED: Gelsolin [Canis lupus familiaris]	gi|545518174	94	1.70
PREDICTED: Fibulin-1 isoform X1 [Canis lupus familiaris]	gi|545514459	78	1.63
Immunoglobulin gamma heavy chain A [Canis lupus familiaris]	gi|17066524	52	1.63
Immunoglobulin gamma heavy chain B [Canis lupus familiaris]	gi|17066526	52	1.61
PREDICTED: EGF containing fibulin-like extracellular matrix protein 1 isoform X3 [Canis lupus familiaris]	gi|57092953	55	1.60
PREDICTED: Angiotensinogen [Canis lupus familiaris]	gi|545494757	52	1.53
Immunoglobulin heavy chain constant region CH2 [Canis lupus familiaris]	gi|124390009	12	1.45
Ig heavy chain V region GOM [Canis lupus familiaris]	gi|123768	12	1.37
Immunoglobulin heavy chain constant region CH4 [Canis lupus familiaris]	gi|124390013	14	1.21
PREDICTED: LOW QUALITY PROTEIN: Serotransferrin isoform 1 [Canis lupus familiaris]	gi|545539001	85	1.16
Protease serine 4 isoform B [Homo sapiens]	gi|33126535	28	1.13
Clusterin precursor [Canis lupus familiaris]	gi|50979240	52	1.08
PREDICTED: Plasminogen isoform X1 [Canis lupus familiaris]	gi|545485785	91	1.06
Immunoglobulin gamma heavy chain D [Canis lupus familiaris]	gi|17066530	51	0.99
EGF-containing fibulin-like extracellular matrix protein 2 precursor [Bos taurus]	gi|115496928	50	0.96
beta-2-Glycoprotein 1 precursor [Canis lupus familiaris]	gi|54792721	38	0.90
PREDICTED: Fetuin-B [Canis lupus familiaris]	gi|74003556	42	0.84
PREDICTED: alpha-2-HS-Glycoprotein [Canis lupus familiaris]	gi|545553759	39	0.82
Kallikrein-6 precursor [Canis lupus familiaris]	gi|308210822	27	0.67

PREDICTED: Transthyretin isoform 2 [Canis lupus familiaris]	gi|57089193	16	0.66
PREDICTED: Apolipoprotein A-I [Felis catus]	gi|410972113	30	0.59
PREDICTED: Antithrombin-III isoform 1 [Canis lupus familiaris]	gi|359320010	52	0.57
Pigment epithelium-derived factor [Canis lupus familiaris]	gi|119637837	44	0.53
Serpin peptidase inhibitor, clade A (alpha-1 antiproteinase, antitrypsin), member 1 precursor [Canis lupus familiaris]	gi|121583756	46	0.53
PREDICTED: WAP, Kazal, immunoglobulin, Kunitz and NTR domain-containing protein 2 isoform 1 [Canis lupus familiaris]	gi|73966420	63	0.51
PREDICTED: Procollagen C-endopeptidase enhancer 1 isoform 3 [Canis lupus familiaris]	gi|73957867	49	0.46
IgA heavy chain constant region [Canis lupus familiaris]	gi|598107	37	0.45
Immunoglobulin heavy chain V region [Canis lupus familiaris]	gi|124389941	15	0.42
Hypothetical protein PANDA_009779 [Ailuropoda melanoleuca]	gi|281354297	68	0.41
PREDICTED: Fibulin-5 isoformX1 [Sus scrofa]	gi|194038270	50	0.41
Apolipoprotein E [Canis lupus familiaris]	gi|3915605	35	0.39
Ig heavy chain V region MOO [Canis lupus familiaris]	gi|123774	13	0.36
PREDICTED: Complement factor B [Canis lupus familiaris]	gi|345778397	86	0.34
PREDICTED: Complement factor D isoform X2 [Canis lupus familiaris]	gi|359322165	28	0.33
PREDICTED: Serum albumin-like isoform 1 [Ailuropoda melanoleuca]	gi|301786252	69	0.32
PREDICTED: N-acetyllactosaminide beta-1,3-N-acetylglucosaminyltransferase [Canis lupus familiaris]	gi|545530694	47	0.32
PREDICTED: Complement C3 [Canis lupus familiaris]	gi|545535669	180	0.32
Carbonic anhydrase 2 [Canis lupus familiaris]	gi|223556019	29	0.30
PREDICTED: Complement C4-A [Canis lupus familiaris]	gi|545520262	193	0.29
Immunoglobulin gamma heavy chain C [Canis lupus familiaris]	gi|17066528	52	0.29
PREDICTED: Vitamin D-binding protein isoformX2 [Canis lupus familiaris]	gi|73975215	53	0.28
PREDICTED: Uncharacterized protein LOC100855594 [Canis lupus familiaris]	gi|545544650	33	0.28
PREDICTED: Insulin-like growth factor binding protein 7 isoform 1 [Canis lupus familiaris]	gi|73975083	29	0.28
PREDICTED: Ectonucleotide pyrophosphatase/phosphodiesterase family member 2 isoform X8 [Canis lupus familiaris]	gi|545520671	109	0.28
PREDICTED: Kininogen-1 isoform X3 [Canis lupus familiaris]	gi|545553768	44	0.28
Albumin precursor [Capra hircus]	gi|193085052	66	0.27
Serum albumin precursor [Bos taurus]	gi|30794280	69	0.27
PREDICTED: Fibrinogen beta chain isoform X3 [Canis lupus familiaris]	gi|545524893	56	0.26
PREDICTED: Plasma serine protease inhibitor isoform X3 [Canis lupus familiaris]	gi|57090343	46	0.25
PREDICTED: Histidine-rich glycoprotein isoform X1 [Canis lupus familiaris]	gi|545553762	62	0.25
PREDICTED: Insulin-like growth factor binding protein 6 isoform 3 [Canis lupus familiaris]	gi|73996265	25	0.23
PREDICTED: Kininogen-1 isoformX2 [Canis lupus familiaris]	gi|57109938	48	0.23
PREDICTED: LOW QUALITY PROTEIN: Extracellular matrix protein 1 [Canis lupus familiaris]	gi|345782613	61	0.23
PREDICTED: Ig lambda chain V-I region BL2 [Canis lupus familiaris]	gi|545544658	15	0.21
Ig heavy chain variable region, VH3 family [Homo sapiens]	gi|33319320	13	0.21

Immunoglobulin heavy chain variable region [Mus musculus]	gi|81237613	13	0.21
PREDICTED: Neuronal pentraxin-1 [Canis lupus familiaris]	gi|73964857	47	0.21
PREDICTED: Ganglioside GM2 activator [Canis lupus familiaris]	gi|359319344	22	0.21
PREDICTED: alpha-1B-Glycoprotein [Canis lupus familiaris]	gi|545487024	61	0.20
Immunoglobulin light chain variable region [Homo sapiens]	gi|37694643	12	0.20
PREDICTED: Cystatin-C-like [Canis lupus familiaris]	gi|359322597	16	0.20
PREDICTED: Fibrinogen gamma chain isoform X2 [Canis lupus familiaris]	gi|545524897	50	0.19
PREDICTED: Complement C1r subcomponent isoform X2 [Canis lupus familiaris]	gi|545546515	81	0.18
Albumin, isoform CRA_h [Homo sapiens]	gi|119626071	69	0.18
PREDICTED: Lumican [Odobenus rosmarus divergens]	gi|472392679	38	0.18
PREDICTED: alpha-1-Antichymotrypsin isoform X2 [Canis lupus familiaris]	gi|73964432	47	0.17
Immunoglobulin lambda light chain variable region [Canis lupus familiaris]	gi|164430480	13	0.15
PREDICTED: Soluble calcium-activated nucleotidase 1 isoform X1 [Canis lupus familiaris]	gi|545509197	94	0.15
PREDICTED: Dihydropyrimidinase-related protein 2 isoform 2 [Macaca mulatta]	gi|109085951	74	0.14
PREDICTED: Calsyntenin-1 [Jaculus jaculus]	gi|507547113	108	0.14
PREDICTED: N-Acetyllactosaminide beta-1,3-N-acetylglucosaminyltransferase [Sus scrofa]	gi|311247200	47	0.14
Anti-quaternary epitope monoclonal antibody light chain [Macaca mulatta]	gi|325152604	15	0.14
PREDICTED: Vitamin K-dependent protein S [Canis lupus familiaris]	gi|545552717	117	0.13
PREDICTED: alpha-Enolase [Trichechus manatus latirostris]	gi|471407206	47	0.11
PREDICTED: Uncharacterized protein LOC612122 [Canis lupus familiaris]	gi|545544656	29	0.10
Carbonic anhydrase 1 [Canis lupus familiaris]	gi|223556021	29	0.10
Unnamed protein product [Mus musculus]	gi|12846758	50	0.10
PREDICTED: Vitronectin isoform 2 [Canis lupus familiaris]	gi|73966959	54	0.09
PREDICTED: LOW QUALITY PROTEIN: Monocyte differentiation antigen CD14 [Canis lupus familiaris]	gi|345794218	40	0.09
PREDICTED: Neural cell adhesion molecule 2 [Canis lupus familiaris]	gi|545550881	93	0.09
PREDICTED: Complement C1q subcomponent subunit C isoform 1 [Canis lupus familiaris]	gi|345793701	26	0.09
Matrix metalloproteinase-2 [Canis lupus familiaris]	gi|7688731	71	0.08
Rho GDP-dissociation inhibitor 1 [Bos taurus]	gi|28603774	23	0.08
mCG7547, isoform CRA_c [Mus musculus]	gi|148703743	39	0.07
PREDICTED: Properdin [Canis lupus familiaris]	gi|74007356	51	0.07
PREDICTED: Bisphosphoglycerate mutase isoform X1 [Canis lupus familiaris]	gi|545525542	30	0.06
Ig lambda chain V-I region BL2 precursor-like protein [Camelus ferus]	gi|528761490	32	0.06
PREDICTED: Hyaluronan-binding protein 2 [Canis lupus familiaris]	gi|545547980	62	0.06
PREDICTED: Corticosteroid-binding globulin isoform X2 [Canis lupus familiaris]	gi|73964415	45	0.05
Olfactomedin-like protein 3 precursor [Bos taurus]	gi|115497736	46	0.05
PREDICTED: Serum albumin [Gorilla gorilla gorilla]	gi|426344604	69	0.05

PREDICTED: Contactin-1 isoform 12 [Pan troglodytes]	gi|114645302	113	0.05
GDP dissociation inhibitor 2, isoform CRA_a [Homo sapiens]	gi|119606836	48	0.05
PREDICTED: LOW QUALITY PROTEIN: Brevican core protein [Equus caballus]	gi|545216545	99	0.05
PREDICTED: Hyaluronidase-1 isoform X3 [Canis lupus familiaris]	gi|545533633	53	0.04
PREDICTED: alpha-2-Antiplasmin isoform X3 [Canis lupus familiaris]	gi|545512145	62	0.04
Hypothetical protein PANDA_005179 [Ailuropoda melanoleuca]	gi|281348494	66	0.04
PREDICTED: Complement factor I isoform X4 [Canis lupus familiaris]	gi|545552242	67	0.03
PREDICTED: Phospholipid transfer protein isoform X4 [Canis lupus familiaris]	gi|545540927	61	0.02
PREDICTED: Transketolase isoform 1 [Odobenus rosmarus divergens]	gi|472384275	68	0.02
PREDICTED: Fibrinogen alpha chain [Canis lupus familiaris]	gi|73978329	97	0.01
Collagen alpha-1(XVIII) chain precursor [Bos taurus]	gi|134085613	154	0.01
PREDICTED: Coagulation factor V isoform X1 [Canis lupus familiaris]	gi|345803274	250	0.01
VivaPure			
Serum albumin precursor [Canis lupus familiaris]	gi|55742764	69	11.30
Hemoglobin subunit beta-like [Canis lupus familiaris]	gi|399567834	16	5.69
PREDICTED: Apolipoprotein A-I [Canis lupus familiaris]	gi|73955106	30	4.80
PREDICTED: Angiotensinogen [Canis lupus familiaris]	gi|545494757	52	3.35
PREDICTED: Serum albumin isoform X1 [Canis lupus familiaris]	gi|545520919	69	2.98
Hemoglobin subunit alpha [Canis lupus familiaris]	gi|44888810	15	2.91
Immunoglobulin gamma heavy chain B [Canis lupus familiaris]	gi|17066526	52	2.58
PREDICTED: Immunoglobulin lambda-like polypeptide 5-like isoform X1 [Canis lupus familiaris]	gi|545544681	25	1.62
PREDICTED: Immunoglobulin lambda-like polypeptide 5-like isoform X2 [Canis lupus familiaris]	gi|545544683	25	1.62
Prostaglandin-H2 D-isomerase precursor [Canis lupus familiaris]	gi|50978842	21	1.33
Albumin, isoform CRA_h [Homo sapiens]	gi|119626071	69	1.21
Immunoglobulin heavy chain constant region CH2 [Canis lupus familiaris]	gi|124390009	12	1.18
Immunoglobulin gamma heavy chain A [Canis lupus familiaris]	gi|17066524	52	1.16
beta-2-Glycoprotein 1 precursor [Canis lupus familiaris]	gi|54792721	38	1.00
PREDICTED: Gelsolin [Canis lupus familiaris]	gi|545518174	94	0.96
PREDICTED: Fibulin-1 isoform X1 [Canis lupus familiaris]	gi|545514459	78	0.86
Immunoglobulin heavy chain constant region CH4 [Canis lupus familiaris]	gi|124390013	14	0.85
PREDICTED: alpha-2-HS-Glycoprotein [Canis lupus familiaris]	gi|545553759	39	0.84
Immunoglobulin gamma heavy chain C [Canis lupus familiaris]	gi|17066528	52	0.67
Clusterin precursor [Canis lupus familiaris]	gi|50979240	52	0.67
PREDICTED: LOW QUALITY PROTEIN: Serotransferrin isoform 1 [Canis lupus familiaris]	gi|545539001	85	0.65
Apolipoprotein E [Canis lupus familiaris]	gi|3915605	35	0.65

PREDICTED: Apolipoprotein E isoform X5 [Canis lupus familiaris]	gi|545488191	47	0.65
PREDICTED: Serum albumin-like isoform 1 [Ailuropoda melanoleuca]	gi|301786252	69	0.64
Serpin peptidase inhibitor, clade A (alpha-1 antiproteinase, antitrypsin), member 1 precursor [Canis lupus familiaris]	gi|121583756	46	0.57
PREDICTED: Vitamin D-binding protein isoformX2 [Canis lupus familiaris]	gi|73975215	53	0.53
PREDICTED: Tetranectin [Canis lupus familiaris]	gi|545535577	18	0.48
PREDICTED: Procollagen C-endopeptidase enhancer 1 isoform 3 [Canis lupus familiaris]	gi|73957867	49	0.45
Immunoglobulin gamma heavy chain D [Canis lupus familiaris]	gi|17066530	51	0.42
PREDICTED: Antithrombin-III isoform 1 [Canis lupus familiaris]	gi|359320010	52	0.42
Ig heavy chain V region GOM [Canis lupus familiaris]	gi|123768	12	0.41
PREDICTED: EGF containing fibulin-like extracellular matrix protein 1 isoform X3 [Canis lupus familiaris]	gi|57092953	55	0.33
Albumin precursor [Capra hircus]	gi|193085052	66	0.30
PREDICTED: Complement C3 [Canis lupus familiaris]	gi|545535669	180	0.25
PREDICTED: Complement component C7 isoform 1 [Canis lupus familiaris]	gi|73953824	95	0.22
Dickkopf-related protein 3 precursor [Canis lupus familiaris]	gi|548923814	38	0.22
PREDICTED: Complement C4-A [Canis lupus familiaris]	gi|545520262	193	0.22
PREDICTED: Calsyntenin-1 [Jaculus jaculus]	gi|507547113	108	0.22
PREDICTED: Plexin domain-containing protein 2 isoform 2 [Pan troglodytes]	gi|114629664	60	0.21
Hypothetical protein PANDA_009779 [Ailuropoda melanoleuca]	gi|281354297	68	0.18
PREDICTED: alpha-1-Antichymotrypsin isoform X2 [Canis lupus familiaris]	gi|73964432	47	0.18
Kallikrein-6 precursor [Canis lupus familiaris]	gi|308210822	27	0.18
Carbonic anhydrase 2 [Canis lupus familiaris]	gi|223556019	29	0.16
PREDICTED: Vitamin K-dependent protein S [Canis lupus familiaris]	gi|545552717	117	0.16
PREDICTED: Uncharacterized protein LOC100855594 [Canis lupus familiaris]	gi|545544650	33	0.14
Immunoglobulin light chain variable region [Homo sapiens]	gi|37694643	12	0.14
PREDICTED: Complement C1q subcomponent subunit C isoform 1 [Canis lupus familiaris]	gi|345793701	26	0.13
PREDICTED: Serum albumin [Gorilla gorilla gorilla]	gi|426344604	69	0.13
PREDICTED: alpha-1B-Glycoprotein [Canis lupus familiaris]	gi|545487024	61	0.12
PREDICTED: Fetuin-B [Canis lupus familiaris]	gi|74003556	42	0.12
PREDICTED: Uncharacterized protein LOC612122 [Canis lupus familiaris]	gi|545544656	29	0.12
PREDICTED: Vitronectin isoform 2 [Canis lupus familiaris]	gi|73966959	54	0.11
PREDICTED: Transthyretin isoform 2 [Canis lupus familiaris]	gi|57089193	16	0.10
PREDICTED: Fibrinogen beta chain isoform X3 [Canis lupus familiaris]	gi|545524893	56	0.09
PREDICTED: Kininogen-1 isoformX2 [Canis lupus familiaris]	gi|57109938	48	0.09
IgA heavy chain constant region [Canis lupus familiaris]	gi|598107	37	0.07
PREDICTED: alpha-2-Antiplasmin isoform X3 [Canis lupus familiaris]	gi|545512145	62	0.07
PREDICTED: Insulin-like growth factor binding protein 6 isoform 3 [Canis lupus familiaris]	gi|73996265	25	0.06

PREDICTED: Complement factor D isoform X2 [Canis lupus familiaris]	gi|359322165	28	0.06
PREDICTED: N-Acetyllactosaminide beta-1,3-N-acetylglucosaminyltransferase [Canis lupus familiaris]	gi|545530694	47	0.05
EGF-containing fibulin-like extracellular matrix protein 2 precursor [Bos taurus]	gi|115496928	50	0.05
Protease serine 4 isoform B [Homo sapiens]	gi|33126535	28	0.05
mCG7547, isoform CRA_c [Mus musculus]	gi|148703743	39	0.04
PREDICTED: Hyaluronan-binding protein 2 [Canis lupus familiaris]	gi|545547980	62	0.03
ProteoExtract			
Immunoglobulin heavy chain variable region [Mus musculus]	gi|81237613	13	2.39
Hemoglobin subunit alpha [Canis lupus familiaris]	gi|44888810	15	1.64
Serum albumin precursor [Bos taurus]	gi|30794280	69	0.64

[1] Accession number in NCBI database
[2] Molecular weight [kDa]
[3] The emPAI value expresses mass spectrometry-based quantitation of protein abundance and is calculated according to http://www.matrixscience.com/help/quant_empai_help.html .
